# Impending Transition From Pediatric to Adult Health Services: A Qualitative Study of the Experiences of Adolescents With Eating Disorders and Their Caregivers

**DOI:** 10.3389/fpsyt.2021.624942

**Published:** 2021-05-26

**Authors:** Ajantha Nadarajah, Gina Dimitropoulos, Christina Grant, Cheryl Webb, Jennifer Couturier

**Affiliations:** ^1^McMaster University, Hamilton, ON, Canada; ^2^Faculty of Social Work, University of Calgary, Calgary, AB, Canada; ^3^Department of Pediatrics, McMaster Children's Hospital, Hamilton, ON, Canada; ^4^Department of Psychiatry and Behavioural Neurosciences, McMaster University, Hamilton, ON, Canada

**Keywords:** eating disorders, health system transition, pediatric care, adult care, qualitative study

## Abstract

**Background:** There is a dearth of research that identifies pediatric to adult health care transition practices that yield positive outcomes for young people with eating disorders (EDs). Further, adolescent and caregiver perspectives are poorly understood and underrepresented in the literature. The purpose of this study, focused on the impending transition from pediatric to adult health services, was twofold: (a) to identify adolescent and caregiver perspectives of barriers and facilitators of a successful transition for adolescents with EDs; and (b) to understand adolescent and caregiver suggestions of interventions for a successful transition.

**Design/Method:** We recruited five adolescents with EDs who were about to be transferred out of pediatric care as well as their caregivers. We conducted a qualitative study in accordance with the principles of interpretive description. Through conducting semi-structured, in-depth interviews with adolescents and caregivers, we investigated their knowledge about health system transitions and anticipated experiences. We identified participants' perceptions of barriers and facilitators regarding a successful transition, as well as their recommendations to improve the transfer of care.

**Results:** Participants possessed a limited understanding of transition processes despite the fact that they were about to be transferred to adult care. From our analyses, the following themes were identified as barriers during the transition process: re-explaining information to adult healthcare providers, lack of professional support while waiting for uptake into the adult health system, and late timing of transition of care discussions. Both adolescents and caregivers expressed that involvement of parents and the pediatric healthcare team helped to facilitate a successful transfer of care. In addition, participants expressed that the implementation of a Transition Coordinator and Transition Passport would be helpful in facilitating a seamless transfer between systems of care.

**Discussion:** These findings demonstrate a significant gap in the system and highlight the importance of developing interventions that facilitate a successful transition. The themes that emerged from this study can inform the development of interventions to facilitate a coordinated transition from pediatric to adult health services for adolescents with EDs.

## Introduction

Eating disorders (EDs) are highly debilitating conditions that impact approximately one million Canadians (1). Globally, the point prevalence of EDs ranges from 4.6% in America, 3.5% in Asia, and 2.2% in Europe (2). EDs such as anorexia nervosa (AN) and bulimia nervosa (BN) have the highest overall mortality rate of any mental illness (3), and are associated with high rates of self-injurious behaviors, suicide, as well as other comorbidities, if left untreated (4–6).

The peak age of onset for EDs is 15 to 25 years and the average duration of illness is approximately 6 years (7). Amongst critical developmental milestones in adolescence and young adulthood, the maximum risk period for the emergence of these disorders also spans the transition boundary from pediatric health services to adult health services (8). In the United States, adolescents typically transition to adult health services between 18 and 21 years of age and in Ontario, Canada, in accordance with government funding models, young people often exit pediatric care at the age of 18 (9).

Transition is defined as “the purposeful, planned process that addresses the medical, psychosocial, educational, and vocational needs of adolescents and young adults with chronic medical and physical conditions as they move from child-centered to adult-oriented health care systems” (10). Building upon this definition, Singh and colleagues have identified four criteria to determine whether transitions are successful: (1) information transfer; (2) period of parallel care; (3) transition planning; and (4) continuity of care (11). Within transition, transfer of care is defined as the logistical components involved in the adolescent formally moving from pediatric to adult healthcare services (12).

Although healthcare in Canada is delivered through a publicly funded system, distribution of funding to ED programs and structure of treatment programs differ throughout the country (13). Referral-based treatment is often provided in outpatient, inpatient, and day treatment programs within tertiary care hospitals (13). In addition, individuals with EDs can also seek private health facilities specialized in ED treatment (13). Canadian practice guidelines to support clinicians' decision-making about ED treatments for children and adolescents have recently been published (14). Some treatment strategies for ED management include family-based treatment, multi-family therapy, residential treatment, cognitive behavioral therapy, and atypical antipsychotics (14, 15). While best practice guidelines for transitioning individuals with chronic conditions to adult healthcare services exist (16, 17), there are no published recommendations for adolescents with EDs.

This transitional period is particularly critical because late adolescence is associated with risk-taking behaviors and greater vulnerability for other mental health disorders, including depression and substance abuse issues (18). Among other significant life transitions in emerging adulthood, such as starting post-secondary education and living independently, adolescents may also disengage from mental health services at increased rates compared to other age cohorts (8, 19). Although factors contributing to disengagement have been postulated, including disease-specific denial, limited self-advocacy skills, and lack of education about transitions, the perspectives of adolescents with EDs and their families are poorly understood and underrepresented (20–22).

Due to the chronicity of these disorders, individuals with EDs often require management into adulthood (11, 23, 24). However, poor transitional practices and processes can contribute to the disruptions in the continuity of care and may lead to poorer long-term clinical outcomes in these individuals (18, 25).

Several interventions are currently used to help facilitate a successful transition from pediatric to adult health services in other healthcare sectors, however, the literature lacks robust and consistent evidence of their effectiveness. For instance, transition training through a Transition Coordinator have been attempted, however, evidence is based on small, non-random, retrospective studies lacking a control group (26, 27). Health Passports are found to be useful by patients, however, there is a need for larger, longitudinal studies to gain a more comprehensive understanding of their efficacy (28). Although transition readiness and appropriateness measure tools can help highlight barriers to a successful transition as well as identify who should be transitioned to adult health services, further studies within the population of adolescents with EDs are required (29). There also appears to be a lack of consensus on how to measure a successful transition from pediatric to adult mental health services (30).

In addition, evidence of specific pediatric-to-adult health system transition interventions for adolescents with EDs is sparse. For instance, a prospective cohort study by Cappelli and colleagues (31) demonstrated effectiveness of a shared management model for transition practices. However, only 3.6% of the sample consisted of individuals with EDs, demonstrating the need for further intervention development studies pertaining to individuals with EDs.

There have been few previous qualitative studies that investigated perspectives of individuals with EDs. A qualitative study by Dimitropoulos et al. retrospectively identified critical perspectives from adolescents with EDs who had transitioned from the pediatric health system to the adult health system about systemic barriers, including lengthy waitlists, and recommendations for improved transfer of care, such as increased education (22). However, the study noted that results were subject to recall bias of participants and since most participants in the study remained ill, perceptions of transfer of care were discussed to be potentially influenced by their ED (22). Another study by Dimitropoulos et al. investigated adolescents with EDs who had previously transferred from pediatric to adult health services to understand their experiences related to family support during the transitional period (32). Although family members were critical in navigating the transfer of care, the study also discussed that conflicts may arise between young people with EDs and their family members due to factors related to illness and emerging adulthood (32). In addition, a qualitative study conducted by Dimitropoulos et al. identified challenges in transition practices from the perspectives of service providers who worked with individuals with EDs, such as reduced parental involvement (21).

Our study offers a new perspective to the current body of literature by eliciting the perceptions and experiences from young people who are about to be transferred from pediatric care as well as their caregivers. Emerging evidence from the perspectives of adolescents with special healthcare needs demonstrates that heath system transition practices should be more sensitive to the unique needs of adolescents and families (33). Further, expectations of caregiver involvement are often higher for families in pediatric ED programs compared to other health sectors because caregivers are accustomed to participating in family-based ED treatment strategies (21). Thus, the perspectives of both caregivers and adolescents with EDs offer valuable insight about the familial experiences of transitioning from pediatric healthcare services. The findings from this study may contribute to the development of a tailored intervention to aid transitions from the pediatric health system to the adult health system.

The objectives of this study, focused on the impending transition from pediatric to adult health services, were to: (1) identify adolescent and caregiver perspectives of barriers and facilitators of a successful transition for adolescents with EDs, and (2) understand adolescent and caregiver perspectives of interventions for a successful transition.

## Materials and Methods

In accordance with the principles of interpretive description, we interviewed a purposeful sample of adolescents and caregivers; that is individuals who find this research meaningful to them, and considered their experiences in relation to previous knowledge about transitions in healthcare (34). Adolescents and caregivers were qualitatively and independently interviewed. Ethics approval was obtained from the Hamilton Integrated Research Ethics Board.

### Population

We recruited adolescents with EDs as well as family members, which included any individual who provides informal care or caregiving to an individual living with an ED (e.g., parent, partner, spouse, sibling). This study aimed to explore barriers, facilitators, and suitable interventions for pediatric-to-adult health system transitions and thus, we were interested in participants' perspectives as they prepared for the point of transition from pediatric care to adult services. In Ontario, Canada, adolescents typically age out of the pediatric health system at the age of 18 and thereafter, can no longer access pediatric health services.

The inclusion criteria for participation for adolescents were as follows: (a) received a diagnosis of an ED from a licensed psychologist or psychiatrist; (b) currently undergoing treatment for their ED in the pediatric ED program; (c) between 17 and 18 years of age and about to undergo the transition process from pediatric to adult health care services. Eligible participants were also required to speak and understand English.

There are no definitive sample size requirements with respect to interpretive description (34). We recruited a total of 10 participants, including five adolescents and their respective caregivers.

### Data Collection

The interview was conducted in-person in a pre-arranged, confidential, and private conference room at McMaster Children's Hospital, Hamilton, Ontario, Canada. Prior to conducting the interview, the researcher explained the study procedures, answered any further questions about the study, and obtained informed consent from the participants.

Data was collected through the conduct of semi-structured, in-depth interviews, using the guidance of an interview guide (Supplementary File 1). Individual interviews were conducted by the first author, who had no prior relationship to the participants. As interviews progressed, follow-up questions were amended given the content of previous interviews in order to elicit the most comprehensive information possible from the research participants. The interviewer was also able to ask for clarification or encourage participants to elaborate further on their experiences. Ongoing mentorship and consultation with the senior author (JC) were used to ensure that interviews between adolescents and caregivers of the same family were not influenced by one another. Interviews were approximately 30 to 60 minutes in length and were recorded using a digital voice recorder. The interviewer transcribed the audio recording verbatim immediately following the completion of the interview in the confidential and private room where the interview was conducted to protect the data that contained identifying information. The audio recording was immediately destroyed after transcription.

### Data Analysis

Conventional analysis inductively identified pertinent themes related to the perceptions and experiences of pediatric-to-adult health system transition for adolescents with EDs and their caregivers. Summative content analysis provided counts of the barriers, facilitators, and interventions that were identified within stakeholder groups, aiding in the interpretation of the results by demonstrating the themes most relevant for each group, and also provided an indication of the level of importance placed on each barrier, facilitator, and intervention among each group. All transcripts were coded by the principal investigator (AN) and 20% of these transcripts were independently double-coded by an experienced qualitative data coder (JC) to ensure accuracy. Data was managed with the qualitative software NVivo 12.0 (35).

## Results

Ten individuals participated in this qualitative study. There were five adolescents who were medically stable and had received care for their ED at the tertiary pediatric ED program for 1–6 years. The adolescents were all female, with a mean age of 17.4 years (SD = 0.3) and had a history of Diagnostic and Statistical Manual of Mental Disorders, 5th Edition (DSM-5) diagnosis of AN (36). The caregivers were all females and parents to the adolescents. The mean age of the caregivers was 47.3 years (SD = 1.4). Please see Table 1 for more detailed information of the demographic information of the participants.

**Table 1:** Demographic characteristics of study participants.

**Characteristics**	**Number of participants**	**Range (Years)**	**Mean ± SD (Years)**
**Gender**			
Male	0		
Female	10		
**Age**			
Adolescents	5	17.2 −17.9	17.4 ± 0.3
Caregivers	5	46.0 – 49.0	47.3 ± 1.4
**Ethnicity**			
European	8		
Indigenous	2		
**Religion**			
Christianity	7		
Agnostic	1		
Spiritual	2		
**Education Level–Caregivers**			
University/college	5		
**Education Level–Adolescents**			
High school	5		
**Family Annual Income**			
< $20,000	–		
$20,000–$34,999	–		
$35,000–$49,999	2		
$50,000–$79,999	–		
$80,000–$99,999	4		
More than $100,000	4		

*SD, standard deviation*.

Below we outline the themes most frequently reported by participants. Please see Supplementary File 2 for a detailed list of emerging themes.

### Understanding of Transition Processes

The most common themes identified were overall limited understanding about the transition processes and some knowledge about transition processes, specifically about adult healthcare services. These themes are described in further detail below.

#### Theme 1: Limited Understanding About the Transition

Both caregivers and adolescents in this study expressed that their knowledge about the transition to adult healthcare services was limited and often absent, which was more clearly emphasized among caregivers compared to adolescents. For instance, one caregiver expressed:

“*So, when do I start finding out about the transition? Do we just leave here cold turkey and jump to their next appointment? Or is it transitional where you graduate like kindergarten? What does it look like? I have no idea” (C2)*.

Similarly, when an adolescent was asked about her knowledge of the transition process, she simply expressed:

“*Yeah, I don't know much about it” (A1)*.

In this study, only two adolescents and their respective caregivers reported that they had had formal and direct discussions about transition processes with the pediatric healthcare team. In addition, only one of the five adolescents expressed having a formal transition plan for her ED treatment in the adult health system, which included a private adult ED program and was created primarily through collaboration between the pediatric healthcare team and caregiver.

#### Theme 2: Some Knowledge About the Transition

Generally, when participants possessed some information of transition processes, caregivers appeared to be more knowledgeable than adolescents, with a greater understanding of specific adult ED treatment services and plans to initiate contact with these services. When adolescents were aware of transition practices, they discussed general characteristics of adult ED programs, including settings where adult treatment services were offered, such as hospitals and universities.

Notably, both caregivers and adolescents generally possessed negative perceptions of expected services in the adult health system. Participants were uncertain whether adult health services would be able to meet the complex needs of adolescents with EDs and these perceptions appeared to be reinforced by the knowledge of poor experiences of other families. Most caregivers expressed that adolescents would receive better care and be safer in the pediatric health system. The following direct quote reflects this theme:

“*I think it's sometimes, when it's more of an adult situation, it's different. There's not that same level of caring, not so much caring. I think we all have more of that empathy when we see kids in trouble or sick whereas if it was an adult, it is not quite the same I think” (C3)*.

Similarly, adolescents expected that adult treatment services would be different to pediatric treatment services in that adult healthcare providers would be less empathetic, less involved in the adolescent's ED treatment, and more intimidating. The following direct quote reflects this theme:

“*I'm feeling nervous. Because I have heard that when you are an adult, it's kind of like if you want to get better, you get better, but if you don't, then no one is forcing you. So that's just scary” (A4)*.

### Barriers to Transition From Pediatric to Adult Care

Participants in both adolescent and caregiver groups identified several barriers in the upcoming transition from the pediatric health system to the adult health system. Although caregivers and adolescents expressed similar barriers, there were differences in which barriers appeared more frequently in the groups and additional barriers emerged from the caregiver group.

#### Theme 1: Re-explaining and Re-sharing Information to Adult Healthcare Providers

The barrier most frequently cited among adolescent participants was re-explaining and re-sharing information to different adult healthcare providers. In total, three caregivers and three adolescents expressed worry as re-sharing their ED history to different adult healthcare providers would lead to a lack of continuity and completeness in these providers' understanding of the adolescent. For instance, one adolescent expressed the transition to be:

“*Nerve-wracking and tiring. […] It's been so long, so a lot of work to re-explain myself all the time and every time I do, I feel like I'm missing vital information” (A2)*.

Further, the participants discussed that frequently repeating information about the adolescents' medical history was expected to be draining and would feel like the adolescent is beginning the treatment process afresh. For instance, one caregiver expressed:

“*I think it just sometimes can be more daunting just starting all over again, it feels like you're back at the beginning again. […] [If] it's not this constant repetitive thing of having to talk about everything over and over again it would be easier on her especially” (C3)*.

#### Theme 2: Lack of Support While Waiting for Uptake Into Adult Health System

The most frequently discussed barrier among caregivers was having to manage the adolescent's ED independently while waiting to be accepted into adult health services and after being discharged from pediatric health services. Five caregivers and one adolescent in this study expressed this barrier. Notably, caregivers were particularly stressed because they felt ill-equipped to manage the adolescent's mental health crises without support. Emergency departments were discussed to have unrealistically high thresholds for admittance and to provide poor care for the adolescent's ED. In addition, while some caregivers discussed positive experiences with family physicians, others discussed that family physicians lacked skills and knowledge to effectively manage the adolescent's ED. These experiences reinforced fears of lack of support while waiting for admission to a suitable adult treatment facility.

In addition, caregivers identified that if a suitable adult ED management program was not secured prior to the pediatric discharge, caregivers would face the burden of independently navigating the adult mental health system and identifying a suitable adult healthcare provider for the adolescent. For instance, one caregiver expressed:

“*[…] I know it's nice to have somebody. And not be left kind of alone. From just what we're hearing now it might be the case. And so, what do you do in the interim if something like hardship does come up like it often does with this illness? Where does that leave us?” (C5)*.

Further, participants were aware that other structural barriers in the transition process, such as delays between transitioning out of the pediatric ED management program to adult health services due to lengthy waitlists in the adult health system, would contribute to periods where adolescents and caregivers expected to lack ED support. For instance, one adolescent expressed:

“*I also know that it is competitive so I might have to be on a waiting list again, so I have to wait again to get in” (A3)*.

#### Theme 3: Late Timing of Discussions on Transitions

In this study, five caregivers and three adolescents discussed that it was important to feel prepared for the transfer out of the pediatric ED program. As such, these participants expressed that the current timing of transition planning and discussions related to the transition were too late. As most participants in this study reported that they had not yet experienced these discussions with the pediatric healthcare team, they expressed it would be more helpful to begin these discussions at least 1 year before the transfer boundary, when the adolescent turned 17 years old. For instance, one adolescent expressed:

“*Like the beginning of this year, or my birthday when it was a year out. Maybe that would have been a good time” (A4)*.

Similarly, the following direct quote from a caregiver expresses this theme:

“*[…] starting it sooner than later that would be great. And what does it look like moving forward? So that they have time to ponder it, because again they have so much history built on this and it's so emotional. So, I think that the sooner they start learning what it's like, it gives them time to mow over it and start thinking about moving on and what growing up looks like with their ED” (C3)*.

Although caregivers postulated that conversations about transitions may have not occurred yet due to the current treatment needs of the adolescent, earlier transition planning was expected to help participants identify suitable adult ED treatment services and understand the extent to which the pediatric health team will aid the transition process. Notably, these delays in proactive preparation appeared to result in caregivers taking initiative to begin transition planning independently and taking responsibility to contact suitable adult healthcare providers for the adolescent's care. For instance, one caregiver expressed:

“*I would like these conversations to start happening so that we are not scrambling in the last minute. I think that's kind of the feel that I have heard from other families. And that's why we have tried to get ahead of it. We don't want [the adolescent] not to be serviced for 4 months because she's on a waitlist and if that's what's known, I'm surprised and slightly disappointed that we have not had the conversation yet” (C4)*.

### Facilitators to Transition From Pediatric to Adult Care

Participants expressed that there are several facilitating factors that would help to ensure a successful transition from pediatric to adult health services. Both adolescents and caregivers mentioned overlapping facilitating factors and caregivers mentioned several additional facilitators that were absent in the adolescent group.

#### Theme 1: Parental Involvement

Among all participants in this study, parental involvement in the transition process was one of the most frequently expressed facilitating factors. Notably, uncertainty surrounding the level of involvement of parents was expressed as a concern among several participants in this study. While balancing autonomy and the need for parental involvement was discussed among participants, most adolescents and caregivers in this study hoped that parents would continue to be involved in the transition from pediatric care as well as in the adult health system.

Adolescents valued parental involvement to assist in the process of researching and identifying appropriate adult ED health services as well as in supporting their ED management. For instance, one adolescent expressed:

“*I think definitely open communication. My mom has been by my side through all of this. So being completely transparent and not hiding anything. Because in the past it is proven to be detrimental” (A5)*.

All caregivers in this study emphasized the importance of parental involvement in the transition process as a facilitating factor. Caregivers expressed that the adolescent may not be ready to independently communicate with staff in the adult health system and manage ED treatment without parental support. For instance, one caregiver expressed:

“*Just advocating for her is a huge one. Finding someone that she connects with and that feels right for her will be where we can help her. Sometimes being her voice when she is not feeling comfortable enough to share some of her concerns or thoughts. I would say driving her everywhere to the appointments and being part of them. […] So, developing a care team for her outside of [the pediatric hospital] and helping her with that” (C4)*.

Caregivers discussed that several of their roles in the transition process, including evaluating the effectiveness of ED management for the adolescent, providing input in the adolescent's ED treatment, researching and identifying suitable adult ED management programs and providers, as well as supporting the adolescent in the transition process and in their ED recovery, would contribute to successful outcomes in the adolescent's transition to the adult health system. However, caregivers specifically also discussed how differences in opinions surrounding ED management between the caregiver and adolescent would serve as a barrier, such as if the adolescent does not wish to seek treatment in the adult health system despite the high medical risk.

#### Theme 2: Involvement of Pediatric Healthcare Team

All caregivers and adolescents in this study expressed that support and involvement of the pediatric health team in the transition process was a facilitating factor. In addition to helping to prepare for the transition, participants discussed that the pediatric health care team's referrals and recommendations of appropriate adult ED management programs were important.

The adolescents discussed that the expertise of the pediatric healthcare team was valuable to ask questions about the adult health system. Notably, adolescents expressed interest in specific appointments with their pediatric healthcare team focused on discussions related to the transition process. As one adolescent expressed:

“*To let [the pediatric clinician] know that I want a few extra appointments and book them as usual. And make a list of a few questions that I have like logistics, what is the involvement my mom, and let her know that it might be just a bunch of random thoughts that need to be out in the open. I think these appointments should happen before the assessment for the other clinic so I can still be at [the pediatric ED program]” (A1)*.

Similarly, caregivers expressed that the professional support network provided by the relationship with the pediatric healthcare team built over the years would allow them to appropriately assist adolescents and families during the transition. One caregiver expressed:

“*I'm hoping that they will have things that they can recommend and maybe referrals to different adult centers or groups are therapies and stuff like that. Hopefully it's not going to be like leave us high and dry I don't think that would really be the case […]” (C3)*.

Although caregivers hoped that the pediatric healthcare team would help to guide adolescents' transition to the adult health system, many caregivers also acknowledged that there may limitations in the pediatric healthcare team's ability to assist, including their workload and time constraints.

### Transition Interventions

To better facilitate a seamless transition from pediatric to adult health services, we asked participants about their perspectives on transition interventions used in other healthcare sectors, additional strategies they believed would help to improve transition practices, and special considerations relevant to adolescents with EDs that were important for the development and implementation of these interventions.

#### A Meeting Among Adult and Pediatric Healthcare Teams

In an ideal transition, it was explained that the pediatric health team and the adult health team would be in close communication with each other to facilitate a seamless transition for the adolescent. This intervention was the only strategy supported by all adolescents and caregivers in this study. Participants discussed that the pediatric healthcare team has extensive knowledge about the adolescent and caregiver and this meeting would allow the adult healthcare team to gain a better understanding of the family. For instance, one adolescent expressed:

“*I think it would be helpful if [the pediatric healthcare team] went to the adult treatment facility to talk about me […]. I think they know me better and they can transfer some of my- what I don't like, like quirks about me that may not be able to be picked up very early on” (A1)*.

In addition, participants discussed that this meeting could help build a rapport and relationship with adult healthcare providers, which would help them feel more comfortable to share and discuss information about their ED treatments with these providers. Participants also expressed that this collaboration would allow the pediatric and adult healthcare teams to jointly develop suitable adult ED treatment strategies using their expertise and knowledge of the adolescent. Both adolescents and caregivers expressed that these meetings would be helpful because the adolescent would not have to repeat their medical histories to different adult healthcare providers, which was previously discussed as a barrier to the transition process by both participant groups.

Both caregivers and adolescents expressed that this meeting may be difficult because of logistical challenges, such as time constraints of clinicians and difficulties of connecting various healthcare providers in one meeting. However, participants expressed that alternative forms of communication, such as written records could be used. Generally, participants believed that the benefits ascertained from these meetings could outweigh potential challenges.

#### Transition Passport

The Transition Passport was described as a customized, wallet-sized card that could be carried by the adolescent. It would give adolescents instant access to their medical information, which can be provided to adult healthcare providers. All adolescents and three caregivers in this study expressed that the Transition Passport would be useful in the transition process. Both adolescents and caregivers expressed that adolescents would not have to explain their medical histories to each new adult healthcare provider. In addition, adolescents expressed that the Transition Passport would be helpful to remember the name of their medications when having to fill out forms and informing adult healthcare providers. For instance, one adolescent expressed:

“*That would sound good because often when you go to places it depends on what you're doing, but they sometimes ask if you're on any medications and I can never remember what it is and I always say my mom knows and I don't really know it would help me know that kind of stuff” (A2)*.

Participants expressed that a potential challenge with the Transition Passport was that adult healthcare providers might not be able to gain a full understanding of the adolescent as a person using the Healthcare Passport alone. In addition, one caregiver expressed that the adolescent might not carry a wallet-sized card with her, and that a digitalized version would be more beneficial, such as the MyTransition phone application (37). Other participants expressed hesitancies surrounding the use of phone applications, citing concerns of security and the lack of personalization to the adolescent's needs and preferences.

#### Transition Coordinator

The Transition Coordinator was described as a healthcare professional who is well-versed with the pediatric and adult health system, serving as a liaison between both healthcare teams and answering questions that patients and families may have about the transition.

All caregivers and two adolescents in this study expressed that the role of the Transition Coordinator would help to facilitate a successful transition between pediatric and adult health systems. Both adolescents and caregivers discussed that the Transition Coordinator would be helpful in informing them about necessary preparations for the health system transition, navigate the adult mental health system, identify different treatment options that might be suitable for the adolescent, and answer questions related to the transition process. For instance, one caregiver expressed:

“*The coordinator I thought was nice because they can [liaise] between both systems and help you answer any questions because after spending so much time here you are so much more comfortable here. […] In the beginning, it's little bit challenging. It's so many doctors coming on board, […] so that I could see to be quite helpful someone who knows both sides of the spectrum” (C2)*.

Participants expressed additional roles that the Transition Coordinator could take on, such as assisting with mental health emergencies and serving as the point of contact between the adolescent and the various health providers that may be involved with the adolescent's ED treatment in the adult health system.

Adolescents also expressed concern about the role of the Transition Coordinator. For instance, two adolescents discussed that they would not feel comfortable sharing information with a health professional who is introduced at this point of their transition process. As one adolescent expressed:

“*I don't know about the Transition Coordinator. I feel like that would be helpful for other people, but I am more shy and it takes a long time for me to warm up to someone. I feel like if I was to be introduced to another person right now, I don't think it would be helpful for me at this time” (A1)*.

Further, they discussed that the Transition Coordinator would need to have a long-term relationship with the adolescent in order to suggest suitable adult health services. Similarly, another adolescent expressed that she did not believe another health professional would be required in her transition, and that she could successfully manage her transition independently.

#### Other Recommendations

Participants in this study mentioned a number of additional recommendations that would help to achieve a successful transition to adult care. Generally, these recommendations aimed to ensure greater preparation among participants for the transfer of care from pediatric to adult health services. Most frequently, both adolescents and caregivers mentioned that phasing out pediatric healthcare services could improve transition processes. Participants mentioned that phasing out pediatric health services, with periods where the adolescent could access health services from both the pediatric and adult health system, until care was completely transferred to the adult health system, could help alleviate feelings of abrupt withdrawal of pediatric health services. In addition, phasing out healthcare services was discussed to allow adolescents to have an opportunity to build relationships with adult healthcare providers with the ability to discuss these experiences with pediatric healthcare professionals, where they often felt more comfortable. For instance, one adolescent expressed:

“*It takes a lot for you to just tell a random person your problems. So, you have some appointments with [adult healthcare providers] and my therapist now, so just dipping my feet in the water with them and keep going to them more and this therapist less and then eventually full-time there and not them here” (A4)*.

Similarly, other strategies mentioned by the participants were education about the adult mental health system, a booklet with information pertaining to transition processes, and extending pediatric health services beyond the adolescent's 18th birthday. While the participants appeared to be well-versed with the pediatric mental health system, they possessed little knowledge of the adult health system, including differences in care philosophies and available health services. Notably, participants expressed that information about transition practices and adult health system could be delivered in various ways, including booklets and workshops at the pediatric clinic, suggesting that different media for knowledge translation could be useful for adolescents and caregivers.

#### Special Considerations for Implementing Transition Recommendations for Individuals With EDs

Participants mentioned that there were several considerations to be mindful of when implementing interventions for adolescents with EDs. Most frequently, caregivers mentioned that EDs were unique to each adolescent and thus, offering choices of various interventions to adolescents and families could help them choose interventions that were most suitable for their circumstances. Further, caregivers also expressed that the status of the ED could even change from day-to-day, with manageable conditions one day and mental health crises the next day that could not be predicted by the caregiver. Thus, it would be beneficial for a single family to have access to different resources as preferences and needs of the adolescent and family change. For instance, one caregiver expressed:

“*I think it depends on the day for EDs. One intervention that works on one day might not work on another day. […] So, things change from one day to the next, from one meal to the next, so that could definitely be a challenge with EDs because it morphs constantly” (C1)*.

Among adolescents, the most frequently cited concern was that interventions used to aid in transition processes should contain sensitive language and images that would not trigger negative responses among adolescents with EDs. For instance, many adolescents in this study expressed that adolescents with EDs may not want to see their weight on different tools, such as the Transition Passport and phone applications. As one adolescent discussed:

“*I think you should just be careful about what numbers or information you are putting on the card because that can be triggering for some people. And even if it's a healthy weight, it can sometimes get in your own head and you think, ‘I can't believe I weigh this much' ” (A1)*.

### General Themes Arising From Interviews

Throughout the interviews, several general themes emerged from caregivers and adolescents when discussing the upcoming transition to adult health services.

#### Theme 1: Feelings of Stress and Anxiety

All caregivers and adolescents in this study expressed feelings of stress and anxiety related to the transition process. Notably, caregivers expressed a greater number of anxiety-related thoughts and feelings compared to adolescents. For instance, one caregiver expressed:

“*It's new so it feels scary. You want your child to stay well and be well. We've been a part of this hospital for some time, so I just want her to succeed and do well-once we leave here. It is a scary transition […] So this is very scary because you have seen the worst of it. So, what that looks like in the future is scary for sure” (C5)*.

There were various sources that contributed to participants' feelings of anxiety. Among caregivers and adolescents, this theme emerged frequently throughout the interviews when discussing uncertainties surrounding the transition process, the fear of ED relapse, stresses associated with finding a suitable adult healthcare provider, as well as balancing current ED treatment and other life transitions with transition planning.

Among adolescents, similar topics were associated with feelings of anxiety, as well as discussions surrounding greater independence in the adult health system and processes of establishing relationships with new adult healthcare providers. For instance, one adolescent expressed:

“*I think it would be nerve-wracking and very frightening to do these changes” (A2)*.

#### Theme 2: Autonomy and Independence

Among interviews in both adolescent and caregiver groups, although more frequently among caregivers, participants discussed the adolescent's expected increase of independence and autonomy in transitioning to the adult health system. Of particular interest, both adolescents and caregivers expressed that increased autonomy was the most frequently cited factor that contributed to their fear of ED relapse in the adult health system. For instance, one adolescent expressed:

“*Obviously there is a kind of a thrill that comes with independence and staying focused. I think just being focussed on my health and not backtracking because like I said, over the past couple of years, I have made some good progress. But I would hate to see that all go to waste” (A5)*.

Four of the five adolescents in this study expressed that increased autonomy and independence was expected to be challenging and stress-inducing because they would need to learn to take charge of their ED treatment and logistical information surrounding their care. For instance, one adolescent expressed:

“*My mom is here all the time when I'm staying here so when I'm at the adult clinic I won't have my mom come with me I have to be more independent about it and I have to figure it out by myself and be more independent when it comes to that and then I can't rely on her as much so I have to figure it out and try to do it individually” (A3)*.

Although most adolescents appeared anxious about increased autonomy and independence, one adolescent expressed that she was looking forward to it. As the adolescent discussed:

“*I also want to, because I'm turning 18, I want a sense of independence and freedom, and I think I have gotten to a point in the recovery where I can say that I will be able to take care of myself when I'm away at school” (A5)*.

Among caregivers, adolescents' increased autonomy and independence appeared to be challenging as they understood that they would have a lesser locus of control in the adolescents' ED treatment in the adult health system. In addition, caregivers worried that greater autonomy and independence would lead to ED relapse or mental health crises to which they could not intervene. As one caregiver expressed:

“*And your adult child who is essentially the same person the day after their 18th birthday as they are the day before their 18th birthday, but now they have all of this control if they want it and they can make their own choices. As a parent I am fearful of that, her not sticking with it to get better because it is hard. When your parent is the one that is driving you and making you go and making you do that family-based therapy, once you're 18 you do not have to subscribe to that anymore and I think that there is some fear there that things are going to fall […]” (C1)*.

Further, knowledge of adolescents' increased autonomy and independence in the adult health system also contributed to participants' uncertainty about the level of parental involvement in the adult health system.

#### Theme 3: Experiences Entering Into the Pediatric ED Program

Three caregivers in this study frequently drew parallels between their current experiences and their previous experiences navigating the health care system entering into the pediatric ED program and the anticipated transition to adult ED health services. They expressed that the rigid admission criteria and limited number of services in the pediatric health system were sources of anxiety about the anticipated transition to the adult ED program. Caregivers' experiences independently managing the adolescents' ED while waiting for acceptance into the pediatric ED program have seemingly revealed anticipated challenges that were also expected when transferring out of the pediatric ED program. These experiences have revealed several of the identified barriers in this study, including how family physicians are ill-equipped to manage EDs, lengthy waitlists for ED health services, and perceptions of lack of funding for ED treatment services.

Caregivers who discussed positive experiences entering into the pediatric ED program mentioned facilitating factors that they hoped would occur again when transferring to the adult health system, specifically close communication among healthcare providers. As one caregiver expressed:

“*But yes, in an ideal world, that would be great where everybody could communicate. I know that happened coming into the [pediatric ED program]. […] [The pediatrician] knew the [pediatric ED program] team that was helping [the adolescent] and it worked very well-actually, and the transition was actually seamless. […] If I could wish for that moving forward then […] that would happen again where everybody would talk, everyone offered their own opinion” (C1)*.

## Discussion

To our knowledge, this is one of the first qualitative studies to examine the unique perspectives of adolescents with EDs as well as their caregivers immediately prior to the point of transfer from pediatric to adult healthcare services. From our analysis, several pertinent themes emerged.

First of all, a lack of understanding of transition processes among all participants in this study demonstrated a significant gap in the system. Recent studies demonstrate that the point of transition sometimes occurs whether or not clinicians, adolescents, or families are prepared for it (38). Therefore, similar to participants in this study, adolescents with complex and special needs often report a lack of transition knowledge (12) and transitional practices are often interrupted and poorly planned (22, 38, 39). As suggested by caregivers in this study and the literature for adolescents with other special needs, the timing of transfer to adult health services should be based on the adolescent's readiness and ability to navigate the adult health system as opposed to age alone (38, 40). Further, the availability of validated assessment tools to identify the readiness of transition-related skills can help guide healthcare providers, families, and adolescents on readiness for transfer to adult care (40).

While adolescents received most of their information about transition practices from caregivers in this study, adolescents possessed less knowledge about transitions compared to caregivers. This may indicate that healthcare providers and caregivers are not effectively communicating information to adolescents. Further, specific roles and responsibilities of all individuals involved in the transition process may not have been determined and agreed upon. Adolescents may also not be equipped with tools and strategies to manage their health or possess a lack of motivation toward treatment (38), and thus still require caregiver involvement. Once adolescents approach the age of transition, it appears to be beneficial if the pediatric healthcare team and caregivers delivered information about the upcoming transition processes to adolescents using structured and age-appropriate communication, including booklets, workshops, and phone applications, as mentioned by participants in this study. Previous studies suggest that an increased level of preparation about health system transitions may help adolescents develop autonomy and competence, which are associated with positive attitudes about health system transitions and build the foundation for disease self-management skills (41, 42).

Of particular interest, participants in this study discussed negative perceptions of adult health services, which may stem from their limited understanding of the adult health system. As discussed in other studies, negative beliefs about adult health services among participants may demonstrate that transitions are anticipated with fear and anxiety (12). Instead, families should be educated on the differences between pediatric and adult health systems, including the benefits of adult health services in ED treatment, and transition should be discussed as a positive event, such as a graduation (12).

Participants identified several barriers that hinder the transition from pediatric to adult healthcare services, including lengthy waiting lists and continually repeating medical information to different adult healthcare providers. Interestingly, these anticipated barriers were also reported by adolescents with EDs who had transitioned to adult health settings (22), as well as other transitioning adolescents with special health needs (12, 43–45). To reduce these structural barriers, an integrated approach may be required between pediatric healthcare providers and adult healthcare providers, as well as increased funding for ED management programs.

Participants also discussed that the late timing of transition discussions was a barrier. Adolescents and caregivers expressed feelings of anxiety and stress related to uncertainties about transition practices, which is consistent with previous research involving adolescents with EDs who recently transferred from pediatric health services (32), as well as other research examining young adults with mental health disorders (46, 47). The study by Dimitropoulos et al. investigated the experiences of young people with EDs who had already transferred from pediatric to adult health services (32). This study suggests that overall ambivalence about factors, including locus of control for ED management and ED recovery during the transitional process can contribute to feelings of anxiety about the health system transition. Further, the study recommended that discussions surrounding transfer of care from pediatric health services to adult health services should begin as early as possible during treatment in the pediatric health system (32).

As recommended in other healthcare sectors, perhaps discussions about health system transitions should begin when adolescents are between the ages of 12 and 14 years (48). This would enable pediatric healthcare providers to continually acknowledge and support developmental challenges and competing life demands, which were also expressed to be difficult to balance with transition planning by participants in this study. Further, early exposure to discussions surrounding transitions can also help mitigate challenges regarding emotional attachment to pediatric providers by setting clear expectations of future care to mentally equip adolescents and caregivers (48, 49). Despite these advantages, there are implementation challenges for clinicians working with adolescents with mental illnesses, as there is no consensus on who should receive transitional care and how this care should be delivered (50). As mentioned by caregivers as a barrier in this study, it may be uniquely challenging for clinicians to develop transition plans for adolescents entering the pediatric ED program at the age of seventeen, while simultaneously managing the demands of current treatment (50).

Several facilitating factors for health system transitions were identified by caregivers and adolescents. Similar to participants in this study, adolescents with EDs who have already transitioned out of the pediatric health system (21), as well as adolescents with varying health conditions expressed that support from the pediatric healthcare team is integral in navigating the adult health system (51, 52). Further, adolescents with EDs may face unique illness-related factors that interrupt their typical psychological, physiological, and social development. For instance, individuals with ED may experience impairments in cognitive functioning or bone health due to factors associated with their ED, such as low body weight (21, 24, 53, 54). The pediatric healthcare team may play an important role in aiding adolescents' navigation of services and resources to support illness-related factors and comorbidities of their ED in adulthood.

Next, participants' discussions about the importance of parental involvement in the transition process also revealed the complexity of balancing adolescents' autonomy and the need for parental involvement. Participants in this study were distressed by the adolescent's increased autonomy in the adult health system, a finding echoed in other studies (55, 56). Although healthcare providers cannot expect adolescents to be fully autonomous and independent at 18 years, the need for high degree of parental involvement discussed by adolescents and caregivers in this study may suggest that adolescents should be better prepared for their increased responsibility in adult health services. Further, greater shifts in responsibility of care to adolescents prior to transfer to adult health services could improve adjustment to new roles in the adult health system (48).

While encouraging adolescents to take greater control in managing their ED is an important part of development (56), previous studies involving adolescents with EDs and other mental health difficulties also suggest that continued parental involvement in the adolescent's treatment process, regardless of their age, could be beneficial (21, 57). Caregivers in this study more frequently expressed feelings of anxiety and stress related to adolescents' increased autonomy compared to adolescents, demonstrating the degree to which caregivers were impacted by the expected shifts from integral components of the adolescent's care to taking on peripheral roles. These findings indicate that caregivers also require support and education to learn how to slowly disengage from active management roles to more supportive roles in the adolescent's ED treatment. Further, pediatric and adult healthcare teams should collaboratively identify strategies to promote autonomy and self-management skills in adolescents while involving caregivers and addressing the ego-syntonic nature of the illness. In studies with transition-age adolescents with diabetes, continued caregiver involvement was beneficial with gradual, supported, and negotiated handover of responsibilities to adolescents for self-management (58).

Participants discussed their perspectives of several interventions to improve transition practices. First of all, declines in mental health service use after transition could likely be a result of pediatric and adult mental health systems largely functioning as separate entities, with significant differences in approach, eligibility, and care philosophies (19). As participants in our study expressed, it may be valuable for adolescents, caregivers, and healthcare teams to meet together and jointly devise a transition plan to facilitate a seamless transfer of care (59). These meetings may help to alleviate adolescents' and caregivers' lack of trust, fear, and anxiety related to acquiring new adult healthcare providers, as well as promote higher retention rates in adult clinics after the transfer (59). Further, the shift from supportive and long-lasting relationships in the pediatric health system to the unfamiliar environments in adult health services was also expressed among other transition-aged adolescents with complex health needs in other studies (39). Since adolescents expressed that building strong and trusting relationships with healthcare providers was important to them, the opportunity for relationship-building through meetings with pediatric and adult healthcare team members can help adolescents and caregivers adjust to new healthcare providers and adult healthcare environments.

Next, the Transition Passport appeared to be a beneficial tool for adolescents to take ownership and responsibility of their health information as well as enhance communication with adult healthcare providers. Notably, the use of a Transition Passport may help adolescents convey information to new healthcare providers without having to frequently repeat information about their medical history, which was identified as a barrier by adolescents. A Transition Passport may help to facilitate adolescents' understanding of their ED and improve self-efficacy skills, which in turn, could help them make informed decisions about their healthcare and empower adolescents to take more control over their health (60). Further, this study underscored important considerations for developing and implementing a Transition Passport for adolescents with EDs, such as maintaining sensitivity around displays of weight.

Families of adolescents with complex health needs, including EDs, often work with a multiplicity of healthcare professionals. As previous research demonstrates in other health sectors, a Transition Coordinator, with whom adolescents and families can build and maintain a trusting relationship, may be essential to help streamline the transfer to adult health services and support families once transferred out of pediatric health services (27, 52). However, it was notable that adolescents in this study expressed hesitations about the introduction of a Transition Coordinator at this point in their transition trajectory, as the new healthcare professional would lack expertise about the adolescent, and it would be uncomfortable to discuss sensitive and personal issues with them. Perhaps it may be more practical to incorporate the elements of the Transition Coordinator role within existing positions in the pediatric ED program such as social workers or nurses. In other healthcare sectors utilizing Transition Coordinators, such as pediatric diabetes programs, previous evidence has demonstrated this approach to be effective when Transition Coordinators performed direct functions, such as appointment scheduling and follow-up of adolescents who have missed appointments (61).

Finally, recommendations provided by participants to improve health system transitions indicate that educational needs exist for both clinicians and families in preparation for transition. Family physicians are described to be ill-equipped to manage demands of transitions according to participants. In fact, there is little evidence in the literature for how family physicians should be incorporated into transition planning processes for adolescents with mental illnesses (30, 62). Since family physicians play a significant role in supporting adolescents with EDs prior to admission to pediatric ED management programs as well as after transferring out of these programs (63), it is critical to establish specific guidelines surrounding the involvement of family physicians in health system transitions.

In addition, other recommendations for improving transitions that were expressed by participants in our study have been suggested in other healthcare fields for adolescents with special health needs, including commencing adult health service appointments before transitions (64, 65) and allowing the pediatric team to follow-up with the adolescents after they have transitioned into adult care (66).

Overall, these themes indicate that increased and productive collaboration between pediatric and adult health care providers as well as choices of other interventions, including the Transition Passport, based on the needs and preferences of families appears to help mitigate participants' concerns about health system transitions and increase participants' preparedness for transitions. While this study identified several considerations for interventions that were important to adolescents with EDs and their caregivers, further research should include longitudinal and cohort studies to gain a more comprehensive understanding of the suitability, satisfaction, and effectiveness of these strategies to adolescents with EDs and their families.

### Limitations and Future Implications

There are some limitations to this study. First of all, our study included a small sample size of ten participants from one pediatric ED program, and factors pertaining to the region and institution may influence our findings. Furthermore, the sample consisted of participants with similar demographic characteristics, such as ethnicity. Thus, findings may not be representative of the experiences of all caregivers and adolescents with EDs. Finally, participants were recruited through a self-referral process and thus, there may be differences between those who did not choose to participate in the study.

## Conclusion

Our study aims to elevate and amplify the voices of adolescents with EDs and their caregivers who are transitioning from pediatric to adult ED healthcare services. From our study, participants have advocated for significant systemic changes to occur within pediatric healthcare services to better prepare themselves for the impending transition to adult healthcare services. Additional larger qualitative and quantitative studies or mixed method designs are needed to develop and implement appropriate strategies to facilitate a seamless transition for families and adolescents with EDs.

Our study contributes important perspectives of adolescents and caregivers who are about to transition from pediatric health services to adult health services. From the perspective of individuals who are in the process of undergoing the transition, we identified significant gaps of knowledge about transition processes, existing barriers to successful transitions, facilitators of a seamless transition, as well as recommendations for improving this transition. A key finding from this study is that adolescents and their caregivers do not appear to have one collective view about transition. It is important to recognize and acknowledge that a “one size fits all” approach to transition may not be appropriate. Based on the information gathered from this study, strategies and further research should be developed in collaboration with adolescents, caregivers, as well as pediatric and adult healthcare professionals.

## Data Availability Statement

The raw data supporting the conclusions of this article will be made available by the authors, without undue reservation.

## Ethics Statement

The studies involving human participants were reviewed and approved by Hamilton Integrated Research Ethics Board. Written informed consent to participate in this study was provided by the participants.

## Author Contributions

AN along with JC conceived of the idea for this project. AN collected data and drafted the manuscript. AN, JC, GD, CG, and CW contributed to the manuscript by editing and have reviewed the final version. All authors contributed to the article and approved the submitted version.

## Conflict of Interest

The authors declare that the research was conducted in the absence of any commercial or financial relationships that could be construed as a potential conflict of interest.
