# Supplementary material for: Impending Transition From Pediatric to Adult Health Services: A Qualitative Study of the Experiences of Adolescents With Eating Disorders and Their Caregivers
Source: Front Psychiatry. 2021 May 26;12:624942. doi: 10.3389/fpsyt.2021.624942 (PMC8187588; doi:10.3389/fpsyt.2021.624942)
Supplement: Supplementary file 1 [file Table_1.docx]

## Supplementary File 2 – Summary of Emergent Themes

## Table 1. Summary of content areas of focus and emergent themes in adolescent participants

| Content areas of focus  Emergent themes | Number of participants | Number of references across participants |
| --- | --- | --- |
| Understanding of transition processes  Limited understanding of transition processes  Some knowledge of transition processes  Types of services offered in adult health system  Age cut-off for pediatric health services  Negative perceptions of adult health clinic  Source of information about transition processes  Pediatric healthcare team  Caregivers | 5  3  3  3  5  2 | 12  6  4  4  5  2 |
| Barriers of transition process  Re-explaining and re-sharing information in the adult health system  Timing of transition discussions in the pediatric health system are late  Uncertainty surrounding parental involvement  Managing health system transitions with other life transitions (e.g. post-secondary education)  Geographical location of adult health services  Lack of support while waiting for uptake into adult health system  Private nature of adult health services (i.e. financial challenges)  Lack of options and choices for appropriate adult health services for ED management | 3  3  2  2  2  1  1  1 | 7  3  5  4  2  3  1  1 |
| Facilitators of transition process  Involvement of pediatric healthcare team  Parental involvement  Supporting adolescent in ED management  Researching and finding suitable adult ED management services  Feeling prepared for the transition  Specific appointments with pediatric healthcare team to discuss details of transition  Appropriate timing of transition discussions in the pediatric health system  Establishing relationships with adult healthcare providers  Friends and social support | 5  5  5  3  5  5  4  1  1 | 16  13  11  3  14  13  4  1  1 |
| Recommendations of interventions to improve transition processes  *A coordinated meeting among adult and pediatric healthcare teams*  A meeting among adult and pediatric healthcare teams would be beneficial  Associated challenges for a meeting between adult and pediatric healthcare teams  Time constraints of health professionals  Feeling uncomfortable when others speak about the adolescent’s condition  *Transition passport*  A transition passport would be beneficial  *Transition coordinator*  A transition coordinator would be beneficial  A transition coordinator would not be beneficial  *Transition phone application*  A transition phone application would be beneficial  A transition phone application would not be beneficial  *Other recommendations mentioned by participants*  Phasing out pediatric healthcare services  Booklet with information about transition processes  Education on the adult health system  Extending pediatric health services beyond 18^th^ birthday  Implementing more structure in the transition process  *Special considerations for implementing these interventions for individuals with EDs*  Ensuring sensitivity when displaying information related to ED (e.g. weight)  Unable to obtain a comprehensive understanding of the adolescent  General themes arising throughout interviews  Feelings of anxiety and stress  Autonomy  Worry about ED relapse  Feelings of excitement | 5  1  1  5  2  3  3  2  2  1  1  1  1  3  1  5  5  3  2 | 8  1  1  5  2  3  3  2  3  2  1  1  1  4  1  16  11  4  3 |

## Table 2. Summary of content areas of focus and emergent themes in caregiver participants

| Content areas of focus  Emergent themes | Number of participants | Number of references across participants |
| --- | --- | --- |
| Understanding of transition processes  Limited understanding of transition processes  Some knowledge of transition processes  Negative perceptions of adult health clinic  Age cut-off for pediatric health services  Types of services offered in adult health system  Transition processes are difficult    Source of information about transition processes  Pediatric healthcare team  Other parents (e.g. parent support group)  Other healthcare professionals  Media (e.g. television) | 5  3  3  2  2  4  1  1  1 | 26  9  3  3  2  6  2  1  1 |
| Barriers of transition process  Lack of support while waiting for uptake into adult health system  Uncertainty surrounding parental involvement  Finding suitable adult healthcare providers  Timing of transition discussions in the pediatric health system are late  Managing health system transitions with other life transitions (e.g. post-secondary education)  Beliefs that adolescent is not ready to manage ED independently in adult health system  Differences in opinions surrounding ED management between caregiver and adolescent  Managing demands of current ED treatment with future planning  Re-explaining and re-sharing information in the adult health system  Family doctors are ill-equipped to manage eating disorders  Private nature of adult health services (i.e. financial challenges)  Geographical location of adult health services  Perceptions of lack of funding for eating disorder programs  Lack of options and choices for appropriate adult health services for ED management | 5  5  5  5  5  4  3  3  3  2  2  2  2  1 | 16  11  10  6  11  6  10  9  8  5  4  2  2  1 |
| Facilitators of transition process  Parental involvement  Supporting adolescent in ED management  Researching and finding suitable adult ED management services  Providing input in ED management  Judging the effectiveness of ED management  Involvement of pediatric healthcare team  Support in transition process  Recommendations and referrals of suitable adult ED management services  Feeling prepared for the transition  Information from the pediatric healthcare team  Appropriate timing of transition discussions in the pediatric health system  Establishing relationships with adult healthcare providers  Family doctors are helpful in ED management  Having options and choices of available adult healthcare services  Beliefs that adolescent is ready to transition to the adult health system | 5  5  4  1  1  5  4  3  4  4  3  2  2  1  1 | 19  9  5  3  1  13  5  9  9  10  4  5  2  4  1 |
| Recommendations of interventions to improve transition processes  *A coordinated meeting among adult and pediatric healthcare teams*  A meeting among adult and pediatric healthcare teams would be beneficial  A meeting among adult and pediatric healthcare teams would not be beneficial  Associated challenges for a meeting between adult and pediatric healthcare teams  Time constraints of health professionals    *Transition passport*  A transition passport would be beneficial  Neutral or uncertain  *Transition coordinator*  A transition coordinator would be beneficial  *Transition phone application*  A transition phone application would be beneficial  A transition phone application would not be beneficial  *Other recommendations mentioned by participants*  Phasing out pediatric healthcare services  Keeping in contact with youth after they leave the pediatric health system  Education on the adult health system  Extending pediatric health services beyond 18^th^ birthday  *Special considerations for implementing these interventions for individuals with EDs*  Each adolescent’s ED is unique and thus it would be beneficial for families to have options of different interventions  Security and confidentiality of personal information related to ED  Sensitivity surrounding information that are ED triggers (e.g. weight)    General themes arising throughout interviews  Feelings of anxiety and stress  Autonomy  Worry about ED relapse  Comparison of transition to pediatric clinic and pediatric-to-adult health system transition  Communication between former and new healthcare providers  Supportive pediatric healthcare team  Difficulties gaining admission to pediatric healthcare services  Waitlists | 5  1  1  3  2  5  3  2  2  2  1  1  4  2  1  5  5  4  2  2  2  1 | 10  1  1  3  2  10  3  4  2  2  4  2  6  2  1  37  28  6  5  4  4  1 |
