# Supplementary material for: Impending Transition From Pediatric to Adult Health Services: A Qualitative Study of the Experiences of Adolescents With Eating Disorders and Their Caregivers
Source: Front Psychiatry. 2021 May 26;12:624942. doi: 10.3389/fpsyt.2021.624942 (PMC8187588; doi:10.3389/fpsyt.2021.624942)
Supplement: Supplementary file 2 [file Data_Sheet_1.docx]

**Supplementary File 1 - Interview Guide**

*notes in italics are intended for the study interviewer; these comments are not to be read

aloud to the study participant.

**Introduction**

Thank you for agreeing to participate in this interview today. The purpose of this interview is to

learn about your experiences and perspectives on pediatric to adult transitions for adolescents with eating disorders (EDs).

Semi-structured Interview:

**Identification of the Problem**

*The intent of this series of questions is to understand whether the participant recognizes a gap in pediatric to adult health system transition. What are their anticipated experiences and challenges?*

1. Can you tell us about what you know of the pediatric to adult health system transition for adolescents with EDs? Where have you received this information from?
2. From your knowledge, what are some barriers you anticipate with respect to these transitions?
3. From your knowledge, what factors might help facilitate this transition process?

**Facilitating Interventions for Transition**

*In this series of questions, the research team is interested in the best strategies for facilitating transitions out of the pediatric system. The intent of this series of questions is to understand participant perspectives of facilitators in transition or support structures to ease transition processes.*

Various models of transition have been proposed in other areas of health care. Some of these include: the use of a transition co-ordinator, the use of digital media or phone applications, or the use of a passport for the young person making this transition. What do you think about these transition tools and their use with adolescents with EDs? Do you think there could be barriers to the use of any of these tools?

1. Do you think individuals with mental health illnesses such as EDs face special challenges with respect to transition and the use of any of these methods?
2. How do you think parents/caregivers/significant others can be a part of the transition process?

**Healthcare Team Role in Transition**

*The intent of this series of questions is to understand participant perspectives of the extent to which the pediatric and adult healthcare teams should support patients and families.*

An ideal transition is proposed to involve the old team meeting with the new team, a transfer of written and verbal information about the individual and a period of parallel care for the individual.

1. If you have ever observed such a transition, can you describe it? If not, what do you anticipate such an experience to feel like?
2. In your opinion, do you think such a transition would be possible?
3. What barriers exist to implementing these principles within our health care system?
4. Do you have any thoughts on how these barriers could be overcome?
5. Do you have any other thoughts on how more successful transitions could occur?

**Is there anything else you would like to share about the transition process?**

**Participant Demographics**

*Please find out about the participant’s demographics - i.e., Participant’s age, race/ethnicity, gender, socioeconomic status, etc.*

Now we would like to complete the interview by asking you some basic questions about yourself:

1. How old are you? Please provide the month and year of birth.
2. What gender would you identify yourself with?
   1. Male, female, other (please specify)
3. Please indicate which of the following ethnic groups you belong to:
   1. European, South Asian (India, Sri Lanka, Pakistan, Nepal, Bangladesh), Chinese (China, Hong Kong, Taiwan), Other Asian (Japan, Korea, Malaysia, Papua New Guinea), Black African, Coloured African (Sub-Saharan African), Native North/South American, Latin American (Latino), Thailand, Philippines, Vietnam, Arab, Persian, Other (ethnic group not listed)
4. Please specify your religion
   1. Christianity, Sikhism, Traditional Chinese, Hinduism, Judaism, Atheist/Agonistic, Islam, Buddhism, Other
5. Please specify your education level
   1. High school or less, trade school, college graduate, postgraduate education
6. Please specify your total family annual income
   1. Less than $20,000; $20,000-$34,999; $35,000-$49,999; $50,000-$79,999; $80,000-$99,999; More than $100,000

Thank you for your time in completing this interview.
